# Chronic testicular pain after routine hydrocelectomy cured with epidural injection

**DOI:** 10.1016/j.eucr.2024.102829

**Published:** 2024-08-22

**Authors:** Om V. Sakhalkar, Dipen S. Mehta, Bradley A. Morganstern

**Affiliations:** Department of Urology, Medical College of Georgia at Augusta University, Augusta, GA, USA

Chronic orchialgia is testicular pain lasting more than 3 months, and hydrocelectomies could cause chronic orchialgia. This patient's orchialgia resolved after administration of an epidural injection four months after his hydrocelectomy. This case report would be the fourth known case of an epidural injection used to treat chronic orchialgia in a pediatric patient, and likely the first known case of an epidural injection used to treat orchialgia after a hydrocelectomy in a pediatric patient. This case outlines unsuccessful and successful treatments of pediatric chronic orchialgia in a post-operative context and could contribute to future guidelines in chronic orchialgia management.

## 1 Introduction

Chronic orchialgia is defined as testicular pain that lasts more than 3 months, significantly interfering with an individual's daily activities.^1^ Most patients are men in their 30s. Etiologies for chronic orchialgia include surgeries like hernia repair or vasectomy and diagnoses like varicocele, hydrocele, and epididymitis.^1^

In this case, we describe a 17-year-old male patient with chronic orchialgia after undergoing a hydrocelectomy. Hydrocelectomies, though common, can result in complications such as recurrence, infection, and chronic orchialgia.^2^ This patient's orchialgia resolved after administration of an epidural injection four months after his hydrocelectomy. To our knowledge, this is the first reported case of this treatment being used to treat testicular pain after hydrocelectomy in a pediatric patient.

## 2 Case

**Fig. 1:**
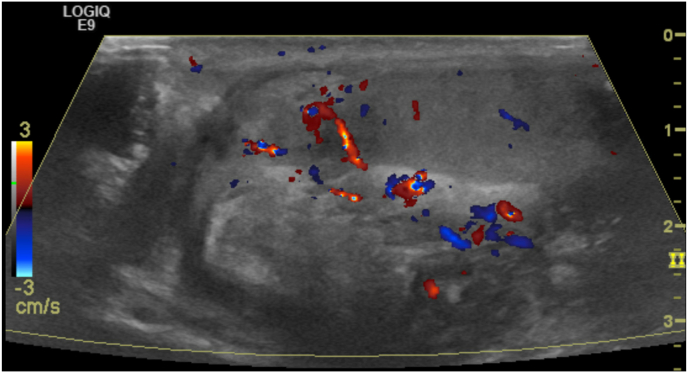
Ultrasound of left testicle

**Fig. 2:**
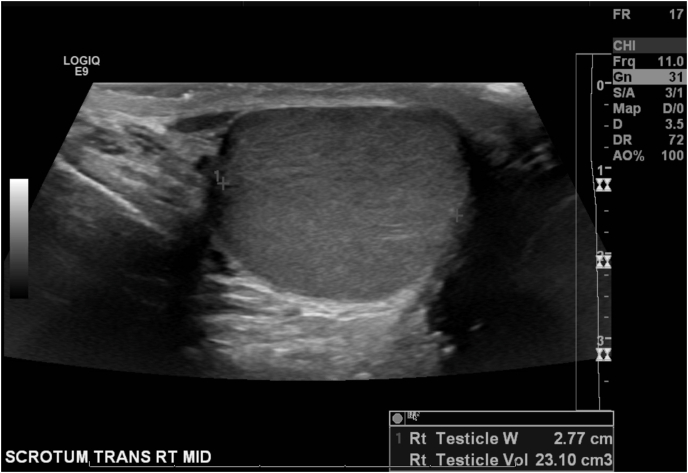
Ultrasound of right testicle

A healthy 17-year-old male underwent a left hydrocelectomy after being diagnosed with a noncommunicating hydrocele. The patient tolerated an uncomplicated Jaboulay hydrocelectomy well with no immediate complications. However, three weeks later, the patient underwent a scrotal ultrasound due to increased swelling and “pressured” pain (Fig. 1, Fig. 2). Both testes were located in the scrotum, with the right testis measuring 23.1 mL and the left testis measuring 22.4 mL. Two small cysts were identified in the left epididymis, with a mild right-sided varicocele. There was no testicular torsion. He was given 500 mg of cephalexin twice a day five days before his ultrasound, but after his ultrasound, he was stopped on cephalexin.

About two weeks later, the patient underwent scrotal exploration because of continued swelling and inflammation. The testicle and cord appeared congested. Stitches from the previous Jaboulay closure along the posterior testicle and cord were released, after which the testicle appeared less congested. A Penrose drain was placed in the dependent portion of the scrotum and secured the drain with a 3-0 silk suture. 0.2% ropivacaine was injected for local wound anesthesia, and a cord block was performed.

Three weeks later (and two months after his first hydrocelectomy) he reported continued pain and swelling on the left scrotum. Pain was reported to be worse with sitting or standing in a shower when he has no scrotal support. He reported continued left scrotal swelling but denied any fever. The pain following the initial procedure was described as pressure in his left scrotum, whereas the pain following the second procedure was described as stinging and burning in the left scrotum. Initially following the second procedure he had pain in his left testes/scrotum with voiding, but this resolved. He had been taking ibuprofen and acetaminophen while wearing supportive underwear instead of the provided jock strap.

As a result, he underwent a spermatic cord block, with 4.5 mL of 0.25% bupivacaine, 4.5 mL of lidocaine 2%, and 40 mg (1 mL) of Kenalog. The patient noted some immediate relief from the procedure.

Two weeks later, the patient underwent a scrotal ultrasound due to recurrence of his pain. This ultrasound noted no evidence of testicular torsion or inflammation, with stable left epididymal cysts. He underwent a left ilioinguinal nerve block and a spermatic cord block that day. The ilioinguinal nerve block used 5 mL of 0.25% bupivacaine, 5 mL of lidocaine 2%, and 80 mg (2 mL) of Kenalog. The spermatic block used 9 mL of 0.25% bupivacaine, 5 mL of lidocaine 2%, and 40 mg (1 mL) of Kenalog. He was prescribed 300 mg of gabapentin three times daily, 100 mg of doxycycline twice daily, 4 mg of Medrol Dosepak daily, and 0.4 mg of tamsulosin daily.

**Fig. 3:**
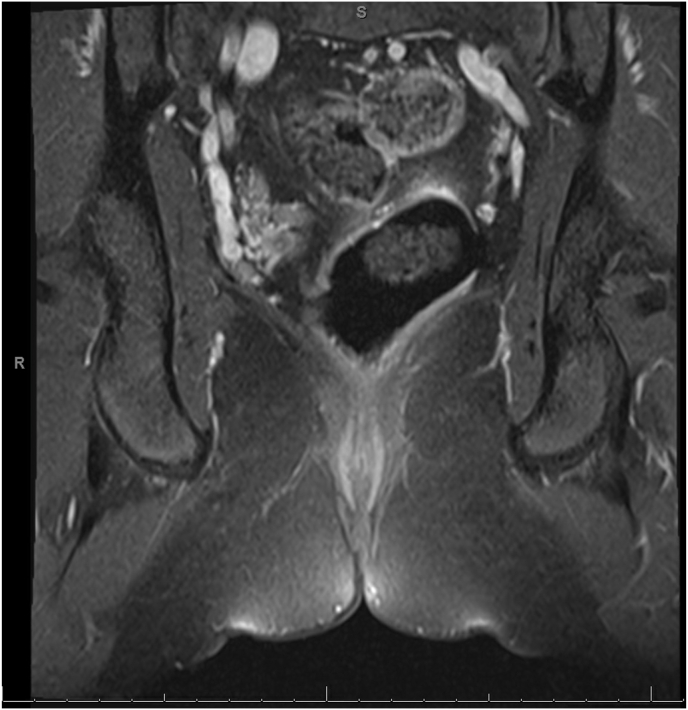
MRI pelvis

Two weeks later, he underwent an additional left ilioinguinal nerve block due to regression of pain 5 days after his most previous procedures. The ilioinguinal nerve block used 5 mL of 0.25% bupivacaine, 5 mL of lidocaine 2%, and 80 mg (2 mL) of Kenalog. At this point he was only taking gabapentin, doxycycline, and tamsulosin. He denied other lower urinary tract symptoms, hematuria, dysuria, abdominal pain, flank pain, or unintentional weight loss. A CT with IV contrast 2 days later and an MRI of his abdomen and pelvis 3 weeks later were normal (Fig. 3). He was referred to pain management, where he was prescribed 10 mg of amitriptyline every night.

One month later (and four months following initial hydrocelectomy), the patient received an epidural steroid injection from pain management. He was anesthetized with 3 cc 1% Lidocaine. 80 mg of Depo-Medrol was injected and flushed with 4 cc of 0.9% normal saline and 2 mL of 0.25% bupivacaine.

This injection led to a complete resolution of pain. He was on 50 mg amitriptyline QHS and 300 mg gabapentin TID during the time period of his epidural injection. He was weaned off to 200 mg gabapentin TID one month later.

**Fig. 4:**
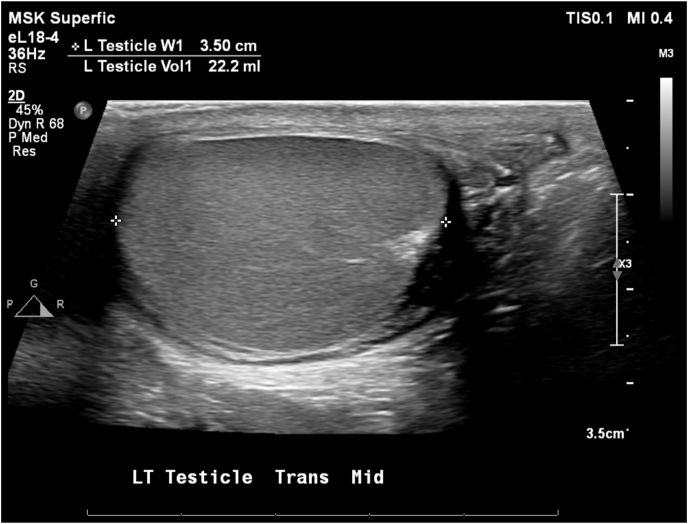
Ultrasound of left testicle

An additional two months later (and seven months after his hydrocelectomy), he reported resolution of his left testicular pain while no longer taking gabapentin and amitriptyline. Scrotal ultrasound showed normal testes bilaterally equal in size (Fig. 4).

## 3 Discussion

Chronic orchialgia is a diagnosis of exclusion with many cases being idiopathic. Thus, management of chronic orchialgia can be difficult and confusing, especially since the pathogenesis can be so varied. Further, literature reporting cases and discussing management of chronic orchialgia in pediatric contexts is scarce.

A thorough review of PubMed found only three cases of an epidural injection being used to treat pediatric chronic testicular pain after conservative management.^3^ This case report would be the fourth known pediatric case of an epidural injection being used to treat chronic testicular pain, and likely the first known case of an epidural injection being used to treat testicular pain after a hydrocelectomy in a pediatric patient. Surgical complications including pain and inflammation are not uncommon after scrotal surgery^4^; however, chronic pain is virtually unreported. Typically, chronic orchialgia management begins with work-up with the goal of finding a source for the pain by taking a complete history, performing a physical exam, and possibly obtaining an ultrasound.^1^^,^^3^ If any possible sources are found, which in this case seemed to be epidydimal cysts and a mild varicocele, surgical exploration would be indicated to resolve the pain. However, with persistence of orchialgia, first-line conservative therapy would involve scrotal support and NSAIDs.^3^ If conservative management fails, the options available for use widely vary and their efficacies are still unclear and debated.^3^ The use of spermatic nerve blocks are temporary pain relief measures that would additionally help predict the efficacy of stronger treatments like microsurgical spermatic cord denervation or, in this case, epidural steroid injections.^1^ S3 transforaminal epidural steroid injections have been shown to be effective at resolving cases of chronic orchialgia due to its action on neuro-axial pain pathways as opposed to just a peripheral block.^3^^,^^5^

We understand that a 17-year-old male patient with a noncommunicating hydrocele and a scrotal hydrocelectomy also fits the description of some adult patients with a similar context. Regardless, this entire case was managed by a pediatric urologist, from the patient's clinical management to the patient's surgical procedures. Urologic conditions affecting patients under the age of 18 are often managed by pediatric urologists, and pediatric urologists are very likely to encounter similar patients with similar characteristics that may require management similar to this patient's management. This case outlines the unsuccessful and successful treatments of pediatric chronic orchialgia in this post-operative context and could contribute to future guidelines regarding management of this condition.

## CRediT authorship contribution statement

**Om V. Sakhalkar:** Writing – review & editing, Writing – original draft. **Dipen S. Mehta:** Writing – review & editing, Writing – original draft. **Bradley A. Morganstern:** Writing – review & editing, Visualization, Validation, Supervision, Software, Resources, Project administration, Methodology, Investigation, Funding acquisition, Formal analysis, Data curation, Conceptualization.
